# Dent’s Disease: A Cause of Monogenic Kidney Stones and Nephrocalcinosis

**DOI:** 10.3390/jpm14060623

**Published:** 2024-06-11

**Authors:** Lucía Diéguez, Melissa Pilco, Sofía Butori, Andrés Kanashiro, Josep Balaña, Esteban Emiliani, Bhaskar K. Somani, Oriol Angerri

**Affiliations:** 1 Department of Urology, Fundación Puigvert, 08025 Barcelona, Spain; sbutori94@gmail.com (S.B.); akanashiro@fundacio-puigvert.es (A.K.); jbalana@fundacio-puigvert.es (J.B.); eemiliani@fundacio-puigvert.es (E.E.); oangerri@fundacio-puigvert.es (O.A.); 2 Department of Nephrology, Fundación Puigvert, 08025 Barcelona, Spain; mpilco@fundacio-puigvert.es; 3 Department of Urology, University Hospital Southampton NHS Trust, Southampton SO16 2HA, UK; b.k.somani@soton.ac.uk

**Keywords:** kidney stones, lithiasis, monogenic, Dent’s disease

## Abstract

Kidney stones are becoming increasingly common, affecting up to 10% of adults. A small percentage are of monogenic origin, such as Dent’s disease (DD). DD is a syndrome that causes low-molecular-weight proteinuria, hypercalciuria, nephrolithiasis, and nephrocalcinosis. It is X-linked, and most patients have mutations in the *CLCN5* gene. We performed a review of the literature and evaluated the case series (*n* = 6) of a single center in Spain, reviewing the natural evolution of kidney stones, clinical implications, laboratory analyses, radiological development, and treatment. All patients had a genetically confirmed diagnosis, with the *CLCN5* mutation being the most frequent (66%). All patients had proteinuria and albuminuria, while only two and three presented hypercalciuria and phosphate abnormalities, respectively. Only one patient did not develop lithiasis, with most (60%) requiring extracorporeal shock wave lithotripsy or surgery during follow-up. Most of the patients are under nephrological follow-up, and two have either received a renal transplant or are awaiting one. The management of these patients is similar to that with lithiasis of non-monogenic origin, with the difference that early genetic diagnosis can help avoid unnecessary treatments, genetic counseling can be provided, and some monogenic kidney stones may benefit from targeted treatments.

## 1. Introduction

Dent’s disease (DD) is a condition caused by a defect in the proximal tubule of the kidney that results in low-molecular-weight proteinuria (LMWP), hypercalciuria, nephrolithiasis (kidney stone formation), and nephrocalcinosis (deposition of calcium in kidney tissue) [1,2]. Its inheritance is X-linked, so males are more frequently affected than females, who may present with a mild phenotype [3]. DD was first described in 1962 by Gentil et al., who presented two cases in children with non-deficiency rickets and tubular dysfunction who could not be included in the classic Fanconi syndrome (aminoaciduria, phosphaturia, bicarbonaturia, metabolic acidosis, glycosuria, rickets or osteomalacia, and growth retardation) [4]. Subsequently, further cases were described (e.g., in 1964 by Dent and Friedman, in 1964 by Scriver et al., and in 1985 by Suzuki et al.) which, although similar, presented phenotypic differences [5]. The name “Dent’s disease” was first suggested in 1990, but it was not until 1999 that the *CLCN5* gene, responsible for the majority of cases, was characterized [6,7]. Although the current prevalence is unknown, the disease has been reported in approximately 250 families [3].

Two genes are currently known to be involved in the pathogenesis of the disease: *CLCN5*, which causes type 1 DD [OMIM 300009], and *OCRL*, which causes type 2 DD [OMIM 300555]. The latter can also present with some extrarenal manifestations, such as mild cognitive impairment, hypotonia, or cataracts, resulting in Lowe syndrome [OMIM 309000]. It is important to note that despite these genetic associations, approximately 25% of patients with clinical manifestations consistent with DD do not have any identified genetic variant [8]. This suggests that other genetic factors or underlying mechanisms may be contributing to the disease in these cases, and further research is needed to fully elucidate the complete genetic landscape of DD.

Monogenic kidney stones are estimated to account for up to 11% of all cases of kidney stones in adults. However, it is possible that the true incidence of monogenic kidney stones is underestimated, as our understanding of this entity is still evolving, and genetic testing may not be routinely performed in all cases [8]. DD is an example of a rare disorder causing monogenic kidney stones.

Our objective in this paper is to analyze the existing literature on this disease, connecting it to the cases of DD found in our urological center, in order to try to determine the best management and follow-up of these patients, as well as the key data for the diagnosis of the disease.

## 2. Materials and Methods

We performed a review of the literature and evaluated the case series of a single center in Spain. Sources used were PubMed, UptoDate, Springerbooks, and ClinicalKey, with “Dent disease”, “Dent syndrome”, “urolithiasis”, “renal calculi”, “kidney stones”, “hypercalciuria”, or “nephrolithiasis” as keywords. Three reviewers (L.D., S.B., and M.P.) selected the articles of the research in the databases previously mentioned. We included studies written in English from 1965 to 2023. To evaluate and describe our case series, we included all the patients followed in our center with a diagnosis of DD genetically confirmed. All patients accepted the use of their clinical data on their first visit to our center. This study provides a detailed analysis of the clinical manifestations and composition of the kidney stones, the treatment approaches, and the disease progression, making comparisons with the currently available information in the literature.

## 3. Results: Dent’s Disease in Our Center

In our center, we recorded six patients who presented to the stone clinic with nephrocalcinosis. The main characteristics of these patients, their clinical presentation, the interventions performed, the treatments received, and the disease evolution are summarized in Table 1. Three of them belonged to the same family: case 2 is a nephew of case 1 and a cousin of case 3. In all cases, the diagnosis was genetically confirmed, with the CLCN5 mutation being the most frequent (66% of patients, *n* = 4). Of the remaining two patients (33%), one had a mutation in OCRL1 associated with Lowe syndrome (case 4). We do not have complete information from the other patient (case 3), who is currently undergoing a new genetic study because the previous one was conducted in another center; however, in this patient, a mutation in CLCN5 is expected because he is a direct relative of other patients affected by this mutation (cases 1 and 2).

Analytically, all patients presented proteinuria and albuminuria, although only two also presented hypercalciuria (cases 1 and 4). None of them had glycosuria or aminoaciduria; one had phosphaturia with hyperphosphatemia; in another, blood phosphate levels were elevated; and one had hypophosphatemia. Half of the patients had microhematuria in the urine sediment. These data are summarized in Table 1 and Table 2. In two patients, cases 4 and 6, a renal biopsy was performed with no specific alterations. In case 4, the biopsy was performed because of abnormalities at urine analysis, while in case 6, it was part of the study of CKD, this being a patient with suspected renal toxicity due to chemotherapy.

Only one of the patients did not develop lithiasis during follow-up, although he presented nephrocalcinosis (case 3). Of the other five patients, one also presented nephrocalcinosis (case 1), and three required one or more treatments for lithiasis. Three patients needed ESWL or surgical interventions, most commonly ureteroscopy, retrograde intrarenal surgery, and endoscopic combined intrarenal surgery. Table 3 shows the treatments performed in these patients and their correlation with renal function. The use of ureteral catheters or nephrostomy tubes was not unusual. All patients underwent imaging tests (CT or US), the frequency varying in accordance with individual needs. In three patients, simple cortical renal cysts were present, being bilateral in two. Regarding the crystallographic study, in four of the patients, the stone composition was mainly calcium phosphate, while in one, it was predominantly calcium oxalate.

Five of the patients (83%) were placed on nephrological follow-up for CKD, and one is currently on the waiting list for renal transplantation (case 3). Case 6 received two renal transplants and finally died because of CKD progression. Three of the patients, those with hypercalciuria or hypercalcemia, are on medical treatment with citrates with or without thiazides, and one of them is also receiving bicarbonate.

## 4. Discussion

DD is a tubulopathy with X-linked inheritance. The true prevalence of the disease is unknown, primarily due to factors such as incomplete penetrance, variable expressivity, and absence of a family history in some affected patients [9]. DD is characterized by altered protein reabsorption and endosomal acidification, which predisposes to proximal tubular pathology, with increased calcium excretion that causes nephrolithiasis and nephrocalcinosis; this leads patients to present chronic kidney disease (CKD), sometimes requiring renal replacement therapy, at an early age [1].

Most of the patients affected by this syndrome have mutations in the CLCN5 gene (Xp11.22), responsible for type 1 DD [1]. The CLCN5 gene encodes a protein of 746 amino acids and 83 kDa, the ClC-5 protein, which is responsible for the regulation of voltage-dependent chloride channels [6]. Up to 148 different mutations in CLCN5 have been described, the most frequent being missense mutations and base substitutions [2]. Ion channels are important for numerous cellular functions, including excitability, synaptic transmission, muscle contraction, transepithelial transport, and cell migration [10]. ClC-5 is found in the subapical endosomes of the proximal tubule and, to a lesser extent, in the loop of Henle and the intercalated cells of the collecting tubule [11]. Here, together with an H^+^-ATPase pump, it is responsible for the endocytic reabsorption of albumin and low-molecular-weight proteins and performs acidification functions [12].

The OCRL1 gene (Xq26) causes type 2 DD and Lowe syndrome, with extrarenal manifestations affecting the brain, with mental and behavioral alterations; the eyes, with congenital cataracts and glaucoma; and the muscles, with growth retardation [13]. Mutations in the OCRL1 gene lead to decreased levels of this protein and inositol polyphosphate 5-phosphatase, which are important for cellular homeostasis. In type 2 DD, only renal manifestations of these mutations are observed, whereas the presence of extrarenal manifestations is indicative of Lowe syndrome. The treatment of these patients is based on early cataract surgery and the management of renal complications [14]. In our series, the patient with the OCRL1 mutation has mild mental retardation and bilateral cataracts that have not yet been treated.

It is estimated that approximately 60% of cases of DD are secondary to mutations in CLCN5 and 15–20% are due to mutations in OCRL1, while in the remaining 25–30%, the mutation remains undetermined, making the diagnosis challenging [5,8,15]. There has even been a suggestion of a third entity, type 3 DD, which would encompass patients with the phenotypic features of DD but without mutations in the aforementioned genes.

In our case series comprising six patients, four have mutations in the CLCN5 gene, and one has a mutation in the OCRL1 gene (case 4). The other patient (case 3) is undergoing another genetic study, but his family has mutations in the CLCN5 gene, so he is very likely to have the same CLCN5 allele. All patients in the series are male, and the majority of them experienced the onset of kidney stones at a young age (an average age of diagnosis of lithiasis of 28 years of age).

Patients with DD have three characteristics: (1) LMWP, which is the earliest and most characteristic finding of the disease; (2) hypercalciuria; and (3) nephrocalcinosis or nephrolithiasis. They may also present hypophosphatemia, rickets, osteomalacia, renal failure, and hematuria [3], but these are not mandatory criteria for diagnosis of the disease, given that in early stages or in women, the disease may present only with LMWP before presenting the full phenotype [16]. In our patients, we consistently observed the presence of LMWP and hypocitraturia. Additionally, some patients exhibited albuminuria, which may be secondary to renal damage, possibly caused by obstructive uropathy or parenchymal injury. Alterations in phosphate, urinary calcium, and blood calcium levels were less common but not infrequent. Importantly, none of the patients displayed clinical data compatible with Fanconi syndrome. Table 2 summarizes the main tubular alterations in our patients.

The accumulation of calcium in the urine is caused by the proximal tubular defect due to mutations in the ClC-5 transporter, which is responsible for reabsorbing most of the calcium from the renal tubule [16]. Defects in this transporter lead to the appearance of nephrolithiasis and nephrocalcinosis, which are usually of calcium oxalate or calcium phosphate [8,17].

**Figure 1:**
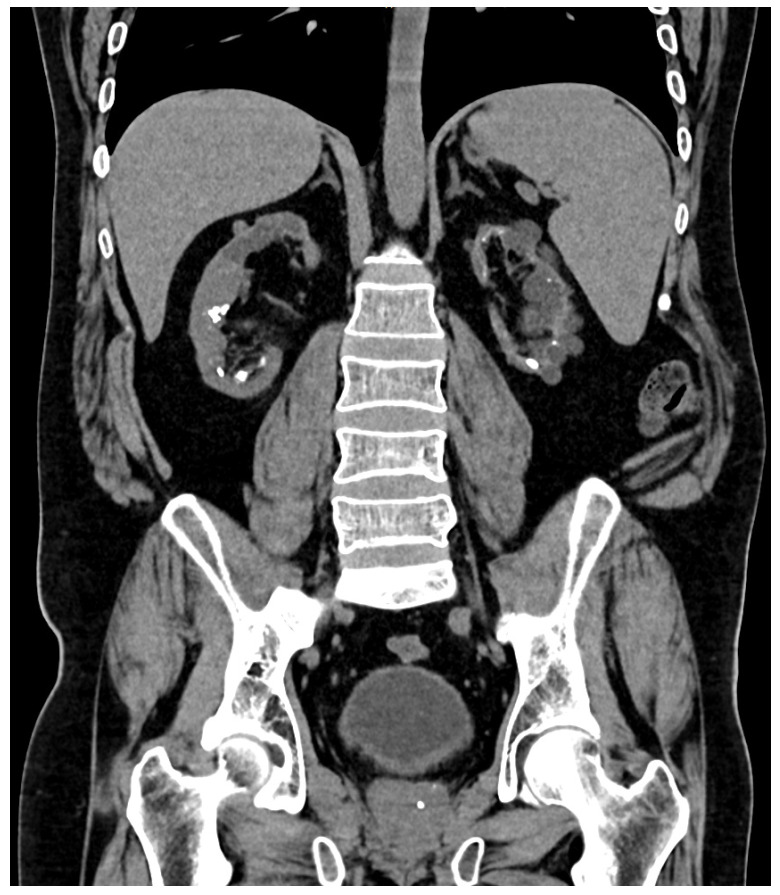
Bilateral lithiasis and microlithiasis in atrophic kidneys in case number 1.

**Figure 2:**
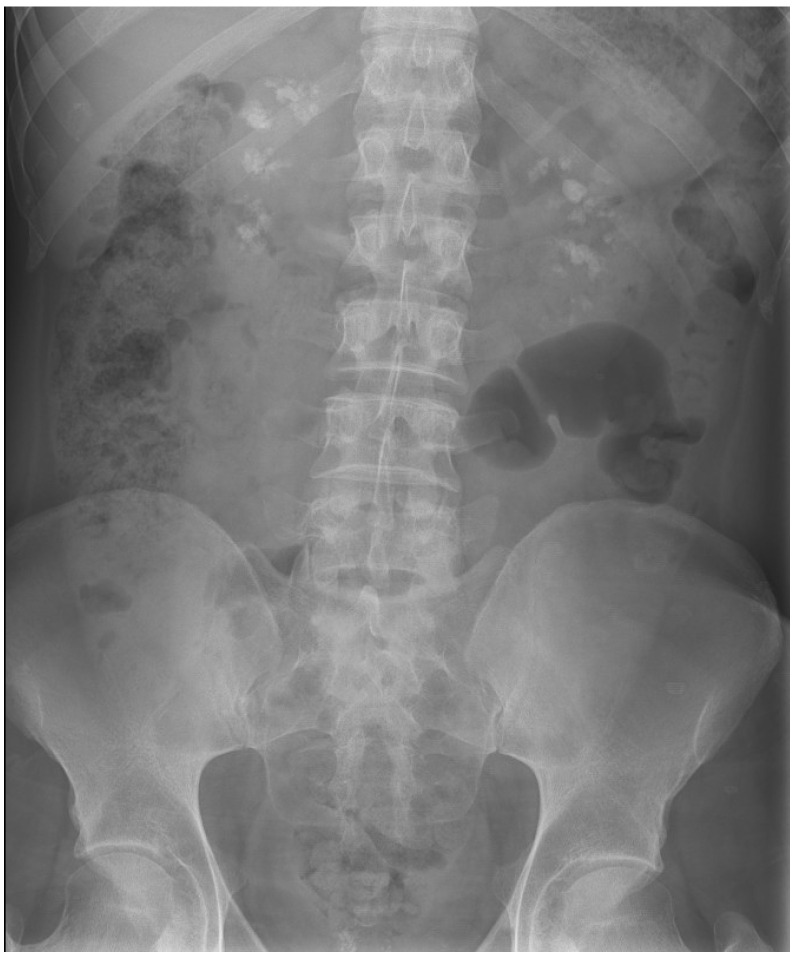
Bilateral lithiasis and microlithiasis in case number 2.

**Figure 3:**
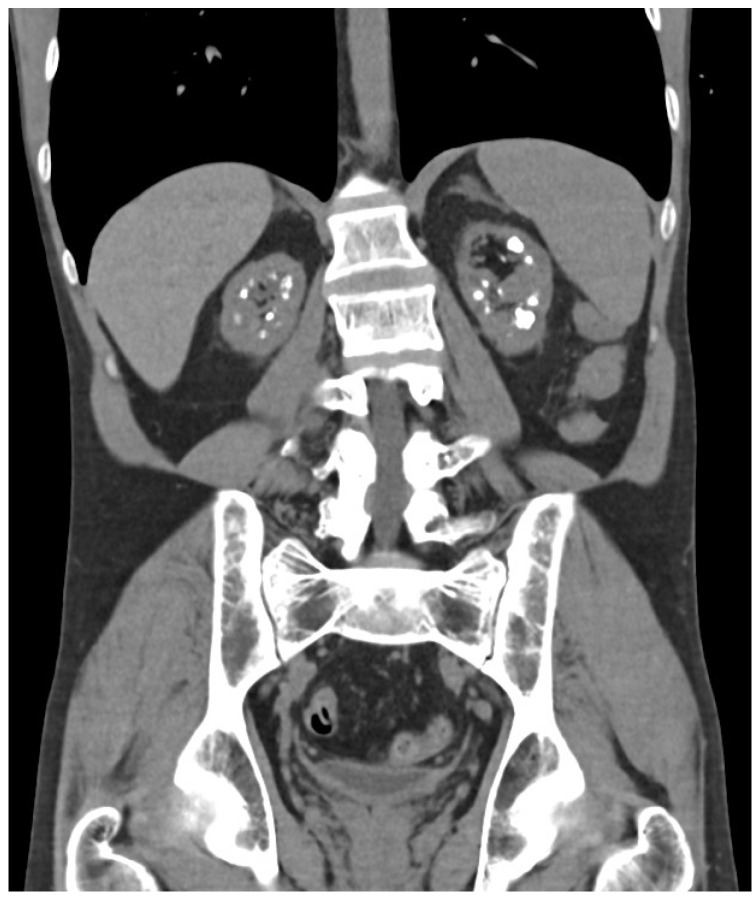
Medullary nephrocalcinosis pattern in case number 3.

The prevalence of urinary lithiasis is progressively increasing, and it is estimated that approximately 5–10% of the general population will present lithiasis at some point in their lives [18]. According to the series studied, up to 11% of kidney stones in adult patients and up to 30% in children have a monogenic origin [8,19]. In DD, concomitant hypercalciuria and kidney stones appear to be more frequent in CLCN5 mutations [12], and hypophosphatemia and hyperphosphaturia are considered independent risk factors for the development of kidney stones and nephrocalcinosis [20]. In our patients, the kidney stones are predominantly composed of calcium phosphate. Four of the six patients developed kidney stones despite undergoing various procedures such as URS, RIRS, ureterolithotomy, ESWL, or ECIRS (Figure 1 and Figure 2), while two remained free of kidney stones at their last radiological follow-up (cases 3 and 5). One patient (case 3) has not had kidney stones during the course of the disease; however, he suffers severe nephrocalcinosis (Figure 3), which has led to end-stage CKD.

CKD is the most important consequence of DD. It usually appears between 30 and 50 years of age, and between 35% and 100% of patients with CKD will develop end-stage CKD [12,17]. To date, the cause of the development of CKD is unknown. It is hypothesized to be related to nephrocalcinosis, but patients without nephrocalcinosis also reach terminal stages of CKD [16]. Furthermore, it is not known whether there is a genotype–phenotype correlation in the severity of lithiasis or nephrocalcinosis, possibly because the number of diagnosed cases is too small to allow such a correlation to be established. In our series, only one of our patients (a young patient) has a normal glomerular filtration rate; most have CKD, one is on the waiting list for a kidney transplant, and one died at an early age as a result of the evolution of his nephropathy.

Clinical suspicion is essential to diagnose kidney stones of monogenic cause. We recommend paying particular attention to those patients with a family history of nephrolithiasis or a history of consanguinity or extrarenal manifestations. Likewise, children with nephrocalcinosis or from families that have had more than one case of nephrolithiasis, especially if these cases have arisen at less than 25 years of age [21], are ideal candidates for tests such as massive DNA sequencing to detect specific mutations. Genetic diagnosis is therefore essential: it allows accurate diagnosis, provision of genetic counseling to patients and their families, and avoidance of unnecessary treatments. Furthermore, genetic diagnosis may lead to access to investigational therapies or novel treatments, offering new avenues for patient care and management.

In addition to basic studies such as blood and urine analysis and ultrasound, abdominal/pelvic CT scans and the analysis of the stone composition are utilized as a complementary technique to provide detailed information about the presence and characteristics of kidney stones and nephrocalcinosis.

As for therapeutic options, there is currently no curative treatment for DD, so the objective is symptomatic control. The main goals of treatment are decreasing hypercalciuria, reducing the incidence of nephrolithiasis and nephrocalcinosis, and delaying the onset of CKD. Among the medical treatments, thiazides are of particular significance as, in conjunction with dietary modifications, they can significantly reduce urinary calcium levels [3]. Hypokalemia is the primary and most concerning side effect observed in our patients, and it often restricts the use of certain treatments. In cases of rickets or osteomalacia, vitamin D or calcium supplementation may be useful, but close monitoring of resulting hypercalciuria is necessary to prevent potential complications. Regarding the treatment of kidney stones, the choice of technique will depend on the location and number of stones. Table 3 summarizes the distribution of treatment for kidney stones in our patients (ESWL or surgical treatment) and the relationship of treatment to renal function. Renal transplantation is reserved for patients with end-stage CKD when other treatment options are no longer sufficient. As DD is a monogenic disorder, the risk of disease recurrence in the transplanted kidney is not considered to be a significant concern. Therefore, renal transplantation can offer a viable and effective treatment option for patients with advanced DD-related CKD, providing them with the potential for improvements in renal function and overall quality of life. However, ongoing monitoring and follow-up care remain crucial to ensure the long-term success of the transplantation and to manage any potential complications that may arise.

## 5. Conclusions

Although rare, DD is a condition that can have important consequences for patients. Changes in lifestyle habits, genetic counseling, and the study of family members (female and male) of affected patients are essential.

As for the management of lithiasis in these patients, there is no specific or standardized treatment, so management is similar to that of patients with renal lithiasis of a non-monogenic cause but with a more intensive follow-up. It is important to insist on water intake and dietary recommendations, and one must take into account the high risk of recurrence of kidney stones in these patients, the presence of terminal CKD, the bilateral distribution of kidney stones, and the possible need for future dialysis or transplantation.

It is recommended that in patients with recurrent lithiasis at an early age and those with a family history or a history of consanguinity, a genetic study should be carried out, taking into account the fact that hypercalciuria and kidney stones are not consistently present in patients with DD. In addition, there are pathologies such as primary hyperoxaluria in which the analysis of lithiasis in the hands of experts is essential to guide the diagnosis.

## Figures and Tables

**Table 1:** Main characteristics of our patients. M: male. F: female. UTI: urinary tract infection. ESWL: external shock-wave lithotripsy. RIRS: retrograde intrarenal surgery. URS: ureterorenoscopy. ECIRS: endoscopic combined intrarenal. YR: years (onset of lithiasis).

	Case 1	Case 2	Case 3	Case 4	Case 5	Case 6
Age	47	28	35	36	53	-
Age of diagnosis	28	10	10	17	50	39
Gender	M	M	M	M	M	M
Family history of lithiasis	Yes	Yes	Yes	No	No	No
Genetic study	CLCN5 c.655T>C in hemizygosis	CLCN5 mutation	Yes, mutation not available	OCRL1 mutation	CLCN5 c.726 + 1G > A in hemizygosis	CLCN5 c.1820C > G in hemizygosis
Past medical history	No	No	No	Mental retardation Bilateral cataracts Scoliosis	No	Tongue neoplasia: chemotherapy at birth
Symptoms	UTI Renal colic	Renal colic	No	No	Renal colic	No
Laboratory tests	Proteinuria 2.04 g/24 h Albuminuria 0.8 g/24 h Hypercalciuria 6.1 mmol/24 h	Proteinuria 2.03 g/24 h Albuminuria 0.4 g/24 h Hypophosphatemia Hypercalcemia	Albumin/creatinine 250 mg/g	Albumin/creatinine 168 mg/g Protein/creatinine 1246 mg/g Hypercalciuria 10.6 mmol/24 h Phosphaturia + hyperphosphatemia	Albumin/creatinine 263 mg/g Protein/creatinine 638 mg/g	Proteinuria 0.3 g/L
Kidney stones	Yes (27 yr)	Yes (10 yr)	No	Yes (30 yr)	Yes (46 yr)	Yes
Nephrocalcinosis	Yes	No	Yes	No	No	No
ESWL/surgery	4 ESWL 1 Ureterolithotomy 2 RIRS	No	No	1 ESWL 1 URS 1 ECIRS	1 ESWL	No
Catheter	3 Nephrostomy tube 1 JJ catheter	No	No	2 JJ catheter	No	No
Crystallographic analysis	90% calcium phosphate 10% protein	80% calcium phosphate	No	90% calcium phosphate	50% calcium phosphate 50% calcium oxalate	No
Last imaging test	CT scan: bilateral lithiasis and microlithiasis, with nephrocalcinosis	US: bilateral microlithiasis	CT scan: atrophic kidneys with a pattern of medullary nephrocalcinosis	CT scan: microlithiasis	US: no lithiasis	CT scan: microlithiasis
Renal cysts	Bilateral	No	No	Unilateral	Bilateral	Bilateral
Medical treatment	Citrates	Citrates Bicarbonate Thiazides	Dialysis	Citrates Thiazides	No	2 kidney trasplants
CKD stage	4A3	No	5A2	3bA2	4A1	-
Renal trasplant	No	No	Waiting list	No	No	Yes (2)

**Table 2:** Summary of the main tubular changes in the six patients. (+): present. (-): absent.

	DD Type	LMWP	Hypercalciuria	Hypophosphatemia	Glucosuria Aminoaciduria Phosphaturia	Hematuria
Case 1	Type 1	+	+	-	-	+
Case 2	Type 1	+	-	+	-	+
Case 3	-	+	-	-	-	-
Case 4	Lowe syndrome	+	+	-	-	+
Case 5	Type 1	+	-	-	-	+
Case 6	Type 1	+	-	-		

**Table 3:** Distribution of the lithiasis treatments in the six patients and glomerular filtration rate (eGFR, CDK-EPI 2009) at the most recent laboratory test. ESWL: extracorporeal shock wave lithotripsy.

	ESWL (*n*)	Surgical Interventions (*n*)	eGFR (mL/min)
Case 1	0	3	20
Case 2	0	0	85
Case 3	0	0	6
Case 4	1	3	44
Case 5	1	0	24
Case 6	0	0	14
Total	2	6	

## Data Availability

No new data were created or analyzed in this study.

## Abbreviations

DD	Dent’s Disease
LMWP	Low-Molecular-Weight Proteinuria
CKD	Chronic Kidney Disease
GFR	Glomerular Filtration Rate
ESWL	External Shock-Wave Lithotripsy
RIRS	Retrograde Intrarenal Surgery
URS	Ureterorenoscopy
ECIRS	Endoscopic Combined Intrarenal Surgery
M	Male
UTI	Urinary Tract Infection
SLC9A6	Solute Carrier family 9 Member A6
CLCN5	Chloride voltage-gated Channel 5
OMIM	Online Mendelian Inheritance in Man
